# A local glucose-and oxygen concentration-based insulin secretion model for pancreatic islets

**DOI:** 10.1186/1742-4682-8-20

**Published:** 2011-06-21

**Authors:** Peter Buchwald

**Affiliations:** 1 Diabetes Research Institute and the Department of Molecular and Cellular Pharmacology, University of Miami, Miller School of Medicine, Miami, FL, USA

**Keywords:** diabetes mellitus, FEM model, glucose-insulin dynamics, Hill equation, islet perifusion, islets of Langerhans, oxygen consumption, PID controller

## Abstract

**Background:**

Because insulin is the main regulator of glucose homeostasis, quantitative models describing the dynamics of glucose-induced insulin secretion are of obvious interest. Here, a computational model is introduced that focuses not on organism-level concentrations, but on the quantitative modeling of local, cellular-level glucose-insulin dynamics by incorporating the detailed spatial distribution of the concentrations of interest within isolated avascular pancreatic islets.

**Methods:**

All nutrient consumption and hormone release rates were assumed to follow Hill-type sigmoid dependences on local concentrations. Insulin secretion rates depend on both the glucose concentration and its time-gradient, resulting in second-and first-phase responses, respectively. Since hypoxia may also be an important limiting factor in avascular islets, oxygen and cell viability considerations were also built in by incorporating and extending our previous islet cell oxygen consumption model. A finite element method (FEM) framework is used to combine reactive rates with mass transport by convection and diffusion as well as fluid-mechanics.

**Results:**

The model was calibrated using experimental results from dynamic glucose-stimulated insulin release (GSIR) perifusion studies with isolated islets. Further optimization is still needed, but calculated insulin responses to stepwise increments in the incoming glucose concentration are in good agreement with existing experimental insulin release data characterizing glucose and oxygen dependence. The model makes possible the detailed description of the intraislet spatial distributions of insulin, glucose, and oxygen levels. In agreement with recent observations, modeling also suggests that smaller islets perform better when transplanted and/or encapsulated.

**Conclusions:**

An insulin secretion model was implemented by coupling local consumption and release rates to calculations of the spatial distributions of all species of interest. The resulting glucose-insulin control system fits in the general framework of a sigmoid proportional-integral-derivative controller, a generalized PID controller, more suitable for biological systems, which are always nonlinear due to the maximum response being limited. Because of the general framework of the implementation, simulations can be carried out for arbitrary geometries including cultured, perifused, transplanted, and encapsulated islets.

## Background

In healthy humans, blood glucose levels have to be maintained in a relatively narrow range: typically 4-5 mM and usually within 3.5-7.0 mM (60-125 mg/dL) in fasting subjects [1,2]. This is mainly achieved via the finely-tuned glucose-insulin control system whereby β-cells located in pancreatic islets act as glucose sensors and adjust their insulin output as a function of the blood glucose level. Pancreatic islets are structurally well-defined spheroidal cell aggregates of about one to two thousand hormone-secreting endocrine cells (α, β, γ, and PP-cells). Human islets have diameters ranging up to about 500 μm with a size distribution that is well described by a Weibull distribution function, and islets with diameters of 100-150 μm are the most representative [3]. Because abnormalities in β-cell function are the main culprit behind elevated glucose levels, quantitative models describing the dynamics of glucose-stimulated insulin release (GSIR) are of obvious interest [1] for both type 1 (insulin-dependent or juvenile-onset) and type 2 (non-insulin dependent or adult-onset) diabetes mellitus. They could help not only to better understand the process, but also to more accurately assess β-cell function and insulin resistance. Abnormalities in β-cell function are critical in defining the risk and development of type 2 diabetes [4], a rapidly increasing therapeutic burden in industrialized nations due to the increasing prevalence of obesity [5,6]. A quantitative understanding of how healthy β-cells maintain normal glucose levels is also of critical importance for the development of 'artificial pancreas' systems [7] including automated closed-loop insulin delivery systems [8-10] as well as for the development of 'bioartificial pancreas' systems such as those using immune-isolated, encapsulated islets [11-13]. Accordingly, mathematical models have been developed to describe the glucose-insulin regulatory system using organism-level concentrations, and they are widely used, for example, to estimate glucose effectiveness and insulin sensitivity from intravenous glucose tolerance tests (IVGTT). They include curve-fitting models such as the "minimal model" [14] and many others [15-17] as well as paradigm models such as HOMA [18,19]. There is also considerable interest in models focusing on insulin release from encapsulated islets [20-26], an approach that is being explored as a possibility to immunoisolate and protect transplanted islets.

The goal of the present work is to develop a finite element method (FEM)-based model that (1) focuses not on organism-level concentrations, but on the quantitative modeling of local, cellular-level glucose-insulin dynamics by incorporating the detailed spatial distribution of the concentrations of interest and that (2) was calibrated by fitting experimental results from dynamic GSIR perifusion studies with isolated islets. Such perifusion studies allow the quantitative assessment of insulin release kinetics under fully controllable experimental conditions of varying external concentrations of glucose, oxygen, or other compounds of interest [27-30], and are now routinely used to assess islet quality and function. Microfluidic chip technologies make now possible even the quantitative monitoring of single islet insulin secretion with high time-resolution [31]. We focused on the modeling of such data because they are better suited for a first-step modeling than those of insulin release studies of fully vascularized islets in live organism, which are difficult to obtain accurately and are also influenced by many other factors. Lack of vasculature in the isolated islets considered here might cause some delay in the response compared with normal islets in their natural environment; however, the diffusion time (*L*^2^/*D*) [32] to (or from) the middle of a 'standard' islet (*d *= 150 μm) is roughly of the order of only 10 s for glucose and 100 s for insulin (with the diffusion coefficients used here)-relatively small delays. Furthermore, because of the spherical structure, most of the cell mass is located in the outer regions of the islets (i.e., about 70% within the outer third of the radius) further diminishing the roles of these delays.

By using a general approach that couples local (i.e., cellular level) hormone release and nutrient consumption rates with mass transport by convection and diffusion, the present approach allows implementation for arbitrary 2D or even 3D geometries including those with flowing fluid phases. Hence, the detailed spatial distribution of insulin release, hypoxia, and cell survival can be modeled within a unified framework for cultured, transplanted, encapsulated, or GSIR-perifused pancreatic islets. While there has been considerable work on modeling insulin secretion, no models that couple both convective and diffusive transport with reactive rates for arbitrary geometries have been published yet. Most published models incorporating mass transport focused on encapsulated islets for a bioartificial pancreas [20-26]. Only very few [21,24] included flow, and even those had to assume cylindrical symmetry. Furthermore, the present model also incorporates a comprehensive approach to account not only for first-and second-phase insulin response, but also for both the glucose-and the oxygen-dependence of insulin release. Because the lack of oxygen (hypoxia) due to oxygen diffusion limitations in avascular islets can be an important limiting [33] factor especially in cultured, encapsulated, and freshly transplanted islets [27,28,34,35], it was important to also incorporate this aspect of the glucose-insulin response in the model.

In response to a stepwise increase of glucose, normal, functioning islets release insulin in a biphasic manner: a relatively quick first phase consisting of a transient spike of 5-10 min is followed by a sustained second phase that is slower and somewhat delayed [36-39]. The effect of hypoxic conditions on the insulin release of perifused islets has been studied by a number of groups [27,28,34,35], and they seem to indicate that insulin release decreases nonlinearly with decreasing oxygen availability; however, only relatively few detailed concentration-dependence studies are available. Parametrization of the insulin release model here has been done to fit experimental insulin release data mainly from two studies with the most detailed concentration dependence data available: by Henquin and co-workers for glucose dependence [40] and by Dionne, Colton and co-workers for oxygen dependence [27].

**Figure 1:**
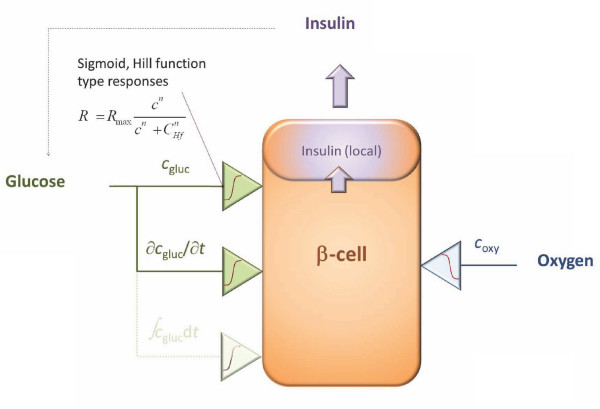
**Schematic concept of the present model of glucose-stimulated insulin release in β-cells**. It is implemented within a general framework of sigmoid proportional-integral-derivative (SPID) controller, and responds to glucose concentrations, but is also influenced by the local availability of oxygen. A total of four concentrations are modeled for 'local' and released insulin (*c*_insL_, *c*_ins_), glucose (*c*_gluc_), and oxygen (*c*_oxy_), respectively.

In the present model, the insulin-secreting β-cells were assumed to act as sensors of both the local glucose concentration and its change (Figure 1). Insulin is released within the islets following Hill-type sigmoid response functions of the local (i.e., cellular level) glucose concentration, *c*_gluc_, as well as its time-gradient, ∂*c*_gluc_/∂*t*, resulting in second-and first-phase insulin responses, respectively. Oxygen and glucose consumption by the islet cells were also incorporated in the model using Michaelis-Menten-type kinetics (Hill equation with *n*_H _= 1). Since lack of oxygen (hypoxia) can be important in avascular islets [33], oxygen concentrations were allowed to limit the rate of insulin secretion using again a Hill-type equation. Finally, all the local (cellular-level) oxygen, glucose, and insulin concentrations were tied together with solute transfer equations to calculate observable, external concentrations as a function of time and incoming glucose and oxygen concentrations.

## Methods

### Mass transport model (convective and diffusive)

For a fully comprehensive description, a total of four concentrations were used each with their corresponding equation (application mode) for 'local' and released insulin, glucose, and oxygen, respectively (*c*_insL_, *c*_ins_, *c*_gluc_, and *c*_oxy_). Accordingly, for each of them, diffusion was assumed to be governed by the generic diffusion equation in its nonconservative formulation (incompressible fluid) [32,41]:(1)

where, *c *denotes the concentration [mol m^-3^] and *D *the diffusion coefficient [m^2 ^s^-1^] of the species of interest, *R *the reaction rate [mol m^-3 ^s^-1^], **u **the velocity field [m s^-1^], and ∇ the standard *del *(*nabla*) operator, [42]. The following diffusion coefficients were used as consensus estimates of values available from the literature: oxygen, *D*_oxy,w _= 3.0 × 10^-9 ^m^2 ^s^-1 ^in aqueous media and *D*_oxy,t _= 2.0 × 10^-9 ^m^2 ^s^-1 ^in islet tissue ([33] and references therein); glucose, *D*_gluc,w _= 0.9 × 10^-9 ^m^2 ^s^-1 ^and *D*_gluc,t _= 0.3 × 10^-9 ^m^2 ^s^-1^; insulin, *D*_ins,w _= 0.15 × 10^-9 ^m^2 ^s^-1 ^and *D*_ins,t _= 0.05 × 10^-9 ^m^2 ^s^-1 ^[23,24]. Published tissue values for glucose vary over a wide range (0.04-0.5 × 10^-9 ^m^2 ^s^-1^) [32,43-46]; a value toward the higher end of this range (0.3 × 10^-9 ^m^2 ^s^-1^) was used here. Very few tissue values for insulin are available (and the existence of dimers and hexamers only complicates the situation) [32,47]; the value used here was lowered compared to water in a manner similar to glucose. For the case of encapsulated islets, the following diffusion coefficients were used for the capsule (e.g., hydrogel matrices such as alginate): *D*_oxy,c _= 2.5 × 10^-9 ^m^2 ^s^-1^, *D*_gluc,c _= 0.6 × 10^-9 ^m^2 ^s^-1^, *D*_ins,c _= 0.1 × 10^-9 ^m^2 ^s^-1 ^[23,48].

### Consumption and release rates

All consumption and release rates were assumed to follow Hill-type dependence on the local concentrations (generalized Michaelis-Menten kinetics):(2)

The three parameters of this function are *R*_max_, the maximum reaction rate [mol m^-3 ^s^-1^], *C*_Hf_, the concentration corresponding to half-maximal response [mol m^-3^], and *n*, the Hill slope characterizing the shape of the response. This function introduced by A. V. Hill [49,50] provides a convenient mathematical function for biological/pharmacological applications [51]: it allows transition from zero to a limited maximum rate via a smooth, continuously derivable function of adjustable width. Mathematically, the well-known two-parameter Michaelis-Menten equation [52] represents a special case (*n *= 1) of the Hill equation, and eq. 2 also shows analogy with the logistic equation, one of the most widely used sigmoid functional forms, being equivalent with a logarithmic logistic function, *y *= *f*(*x*) = *R*_max_/(1 + *βe*^-*n *ln*x*^). Obviously, different parameter values are used for the different release and consumption functions (i.e., insulin, glucose, oxygen; e.g., *C*_Hf,gluc_, *C*_Hf,oxy_, etc.).

#### Oxygen consumption and cell viability

For oxygen consumption, the basic values used in our previous model [33,53] were maintained (*n*_oxy _= 1, *R*_max,oxy _= -0.034 mol m^-3 ^s^-1^, *C*_Hf,oxy _= 1 μM-corresponding to a partial oxygen pressure of *p*_Hf,oxy _= 0.7 mmHg) since, by all indications, the assumption of a regular Michaelis-Menten kinetics (i.e., *n*_oxy _= 1) gives an adequate fit [54,55]. Accordingly, at very low oxygen concentrations, where cells only try to survive, oxygen consumption scales with the available concentration *c*_oxy _and, at sufficiently high concentration, it plateaus at a maximum (*R*_max_). As before [33], to account for the increased metabolic demand of insulin release and production at higher glucose concentrations, a dependence of *R*_oxy _on the local glucose concentration was also introduced via a modulating function *φ_o,g_*(*c*_gluc_):(3)

A number of experiments have shown increased oxygen consumption rate in islets when going from low to high glucose concentrations [56-58]. Here, in a slight update of our previous model [33], we assumed that the oxygen consumption rate contains a base-rate and an additional component that increases due to the increasing metabolic demand in parallel with the insulin secretion rate (*cf*. eq. 6) as a function of the glucose concentration:(4)

Lacking detailed data, as a first estimate, we assumed the base rate to represent 50% of the total rate possible (*φ*_base _= *φ*_metab _= 0.5). To maintain the previously used consumption rate at low (3 mM) glucose, a scaling factor is used, *Φ*_sc _= 1.8. The metabolic component fully parallels that used for insulin secretion (*n*_ins2,gluc _= 2.5, *C*_Hf,ins2,gluc _= 7 mM; see eq. 6 later). With this selection, oxygen consumption increases about 70% when going from low (3 mM) to high glucose (15 mM)-slightly less than used previously in our preliminary model [33], but in good agreement with the approximately 50%-100% fold increase seen in various experimental settings [35,36,56-60]. As before [33], a step-down function, *δ*, was also added to account for necrosis and cut the oxygen consumption of those tissues where the oxygen concentration *c*_oxy _falls below a critical value, *C*_cr,oxy _= 0.1 μM (corresponding to *p*_cr,oxy _= 0.07 mmHg). To avoid computational problems due to abrupt transitions, COMSOL's smoothed Heaviside function with a continuous first derivative and without overshoot flc1hs [61] was used as step-down function, *δ*(*c*_oxy _>*C*_cr,oxy_) = flc1hs(*c*_oxy _- 1.0x10^-4^, 0.5x10^-4^).

#### Glucose consumption

Glucose consumption, in a manner very similar to oxygen consumption, was assumed to also follow simple Michaelis-Menten kinetics (*n*_gluc _= 1) with *R*_max,gluc _= -0.028 mol m^-3 ^s^-1 ^and *C*_Hf,gluc _= 10 μM [23,24,46]:(5)

These parameter values are draft first estimates only; however, changes in glucose concentrations due to glucose consumption by islets have only minimal influence on insulin release or cell survival because oxygen diffusion limitations in tissue or in media are far more severe than for glucose [55,62]. Even if oxygen is consumed at approximately the same rate as glucose on a molar basis and has a 3-4-fold higher diffusion coefficient (i.e., *D*_w_s used here of 3.0 × 10^-9 ^vs. 0.9 × 10^-9 ^m^2 ^s^-1^), this is more than offset by the differences in the concentrations available under physiological conditions. The solubility of oxygen in culture media or in tissue is much lower than that of glucose; hence, the available oxygen concentrations are much more limited (e.g., around 0.05-0.2 mM vs. 3-15 mM assuming physiologically relevant conditions) [62]. Glucose consumption by islet cells alters the glucose levels reaching the glucose-sensing β-cells only minimally.

#### Insulin release

**Figure 2:**
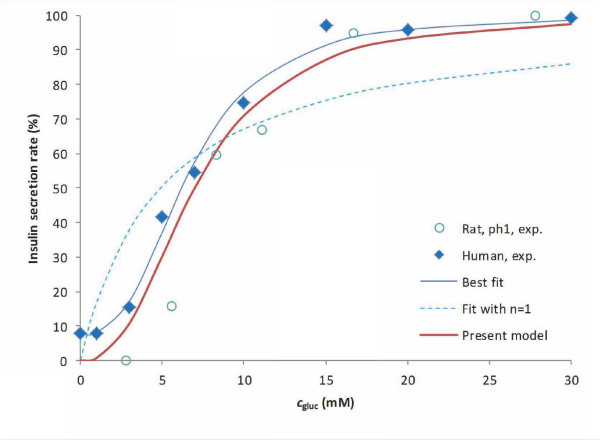
**Glucose-dependence of insulin secretion rate in perifused islets**. Experimental data are for perifused human islets (blue diamonds) [40] and isolated rat pancreas (blue circles) [63]. Fit of the human data with general Hill-type equations (eq. 2) is shown without any restrictions (best fit, *n *= 2.7, *C*_Hf,gluc _= 6.6 mM; blue line), with restricting the Hill slope to unity (*n *= 1, Michaelis-Menten-type function, *C*_Hf,gluc _= 4.9 mM; dashed blue line), and with the present model used for the local concentration (eq. 6) (*n *= 2.5, *C*_Hf,gluc _= 7 mM; red line).

Obviously, the most crucial part of the present model is the functional form describing the glucose-(and oxygen) dependence of the insulin secretion rate, *R*_ins_. Glucose (or oxygen) is not a substrate *per se *for insulin production; hence, there is no direct justification for the use of Michaelis-Menten-type enzyme kinetics. Nevertheless, the corresponding generalized form (Hill equation, eq. 2) provides a mathematically convenient functionality that fits well the experimental results. A Hill function with *n *> 1 is needed because glucose-insulin response is clearly more abrupt than the rectangular hyperbola of the Michaelis-Menten equation corresponding to *n *= 1 as clearly illustrated by the sigmoid-type curve of Figure 2 and by other similar data from various sources [36,40,63,64]. In fact, such a function has been used as early as 1972 by Grodsky (*n *= 3.3, *C*_Hf,ins,gluc _= 8.3 mM; isolated rat pancreas) and justified as resulting from insulin release from individual packets with normally distributed sensitivity thresholds [63]. However, except for some recent work by Pedersen, Cobelli and co-workers [65,66], such a sigmoid functional dependence has been mostly neglected since then, and most models [21,23,24] used flatter (*n *= 1) response functions combined with exponentially decreasing time-functions. To have a model that can be used for arbitrary incoming glucose profiles, the use of explicit time dependency was avoided here; however, use of an additional 'local' insulin compartment with first order release kinetics (see later) achieves a similar effect. A sufficiently abrupt sigmoid response function on *c*_gluc _ensures an upper limit (plateau) at high glucose concentrations as well as essentially no response at low concentrations (Figure 2) eliminating the need for a specified minimum threshold for effect.

Accordingly, the main function used here to describe the glucose-insulin dynamics of the second-phase response is:(6)

with *n*_ins2,gluc _= 2.5, *C*_Hf,ins2,gluc _= 7 mM, and *R*_max,ins2 _= 3.0 × 10^-5 ^mol m^-3 ^s^-1^. These values were obtained here by calculating the predicted insulin output in response to a stepwise increase in incoming glucose and adjusting *n*_ins2,gluc _and *C*_Hf,ins2,gluc _to obtain best fit with the human islet data of Henquin and co-workers (staircase experiment) [40] (Figure 2). Topp and co-workers used a similar Hill function (*n *= 2, *C*_Hf _= 7.8 mM) for insulin secretion based on (rat) data from Malaisse [67]. Compared to rodents, human insulin response is left-shifted, and a half-maximal response for a glucose concentration around 7 mM seems reasonable [40,68]. The activity of glucokinase, which serves as glucose sensor in β-cells and is also generally considered as rate-limiting for their glucose usage, shows a sigmoid-type dependence on *c*_gluc _(i.e., eq. 2 with *C*_Hf,gluc _= 8.4 mM, *n*_gluc _= 1.7 [69] or *C*_Hf,gluc _= 7.0 mM, *n*_gluc _= 1.7 [70]) in general agreement with eqs. 5 and 6 and their parameterization (Table 1). *R*_max,ins2 _corresponds to a maximum (second phase) secretion rate of ~20 pg/IEQ/min for human islets [37,40,71].

**Table 1:** Summary of Hill function (eq. 2) parameters used in the present model (Figure 1, eq. 3-9)

Model	**Var**.	*C*_Hf_	*n*	*R*_max_	Comments
*R*_oxy_, oxygen consumption, base	*c*_oxy_	1 μM	1	-0.034 mol/m^3^/s	Cut to 0 below critical value, *c*_oxy _<*C*_cr,oxy_.

*R*_oxy_, oxygen consumption, *φ*_o,g _metabolic part	*c*_gluc_	7 mM	2.5	N/A	Due to increasing metabolic demand; parallels second-phase insulin secretion rate.

*R*_gluc_, glucose consumption	*c*_gluc_	10 μM	1	-0.028 mol/m^3^/s	Contrary to oxygen, has no significant influence on model results.

*R*_ins,ph2_, insulin secretion rate, second-phase	*c*_gluc_	7 mM	2.5	3 × 10^-5 ^mol/m^3^/s	Total secretion rate is modulated by local oxygen availability (last row).

*R*_ins,ph1_, insulin secretion rate, first-phase	∂*c*_gluc_/∂*t*	0.03 mM/s	2	21 × 10^-5 ^mol/m^3^/s	Modulated via eq. 8 to have maximum sensibility around *c*_gluc _= 5 mM and be limited at very large or low *c*_gluc_.

Insulin secretion rate, *φ*_o,g _oxygen dependence	*c*_oxy_	3 μM	3	N/A	To abruptly limit insulin secretion if *c*_oxy _becomes critically low.

To incorporate a simple model of the first-phase response, we also added a component that depends on the glucose time-gradient (*c_t _*= ∂*c*_gluc_/∂*t*). This is non-zero only when the glucose concentration is increasing, i.e., only when *c*_t _> 0. Again, a Hill-type sigmoid response was assumed to ensure a plateau:(7)

with *n*_ins1,gluc _= 2, *Ct*_Hf,ins1,gluc _= 0.03 mM s^-1^, and *R*_max,ins1 _= 21.0 × 10^-5 ^mol m^-3 ^s^-1^. These parameters are more difficult to directly calibrate from existing data on insulin responses to stepwise glucose increases; hence, they have to be considered as exploratory settings. Constant glucose ramps have been explored with perifused rat islets in an attempt to quantify these responses [72]; however, the gradients used there are too small (1.5-4.5 μM/s) to allow a clear separation between first-and second-phase responses for quantitation. The *Ct*_Hf _value used here (0.03 mM/s) was selected so as to give an approximately linear response for a range that likely covers normal physiologic conditions (e.g., 5 mM increase in 10-20 min: 0.005-0.01 mM/s) as well as dynamic perifusion conditions (e.g., 2-6 mM increases in 1 min: 0.03-0.10 mM/s). A completely linear (i.e., proportional) glucose gradient dependent term has been used in a few previous models mainly following Jaffrin [20,26,72-74] (one of them [73] also allowing modulation of the proportionality constant by glucose concentration). Here, one additional modulating function, *σ*_i1,g _has also been incorporated to reduce this gradient-dependent response for islets that are already operating at an elevated second-phase secretion rate and to maximize it around *c*_gluc _values where islets are likely to be most sensitive (*C*_m _= 5 mM) using a derivative of a sigmoid function:(8)

With all these, total insulin release is obtained as the sum of first-and second-phase releases and an additional modulating function to account for the limiting effect of oxygen availability, which can become important in the core region of larger avascular islets especially under hypoxic conditions:(9)

We assumed an abrupt Hill-type (eq. 2) modulating function as *φ_i,o_*(*c*_oxy_) with *n*_ins,oxy _= 3 and *C*_Hf,ins,oxy _= 3 μM (*p*_Hf,ins,oxy _= 2 mmHg) so that insulin secretion starts becoming limited for local oxygen concentrations that are below ~6 μM (corresponding to a partial pressure of *p*_O2 _≈ 4 mmHg) (Additional file 1, Figure S1). This is a somewhat similar, but mathematically more convenient function than the bilinear one introduced by Avgoustiniatos [75] and used by Colton and co-workers [76] to account for insulin secretion limitations at low oxygen (*p*_O2 _< 5.1 mmHg assumed by them) as it is a smooth sigmoid function with a continuous derivative (Additional file 1, Figure S1).

For a correct time-scale of insulin release, an extra compartment had to be added; otherwise insulin responses decreased too quickly compared to experimental observations (~1 min vs. ~5-10 min). Hence, insulin is assumed to be first secreted in a 'local' compartment (Figure 1) in response to the current local glucose concentration (*R*_ins_, eq. 9) and then released from here following a first order kinetics [d*c*_insL_/d*t *= *R*_ins _- *k*_insL_(c_insL _- *c*_ins_); *k*_insL _= 0.003 s^-1^, corresponding to a half-life *t*_1/2 _of approximately 4 min]. 'Local' insulin was modeled as an additional concentration with the regular convection model (eq. 1), but having a very low diffusivity (*D*_insL,t _= 1.0 × 10^-16 ^m^2 ^s^-1^). Throughout the entire model building process, special care was taken to keep the number of parameters as low as possible to avoid over-parameterization [77]; however, inclusion of this compartment was necessary. The model has been parameterized by fitting experimental insulin release data from two detailed concentration-dependence perifusion studies: one concentrating on the effect of glucose using isolated human islets [40] and one concentrating on the effect of hypoxia using isolated rat islets [27].

### Fluid dynamics model

To incorporate media flow in the perifusion tube, these convection and diffusion models need to be coupled to a fluid dynamics model. Accordingly, the incompressible Navier-Stokes model for Newtonian flow (constant viscosity) was used for fluid dynamics to calculate the velocity field **u **that results from convection [32,41]:(10)

Here, *ρ *denotes density [kg m^-3^], *η *viscosity [kg m^-1 ^s^-1 ^= Pa s], *p *pressure [Pa, N m^-2^, kg m^-1 ^s^-2^], and **F **volume force [N m^-3^, kg m^-2 ^s^-2^]. The first equation is the momentum balance; the second one is simply the equation of continuity for incompressible fluids. The flowing media was assumed to be an essentially aqueous media at body temperature; i.e., the following values were used: *T*_0 _= 310.15 K, *ρ *= 993 kg m^-3^, *η*= 0.7 × 10^-3 ^Pa s, *c*_p _= 4200 J kg^-1^K^-1^, *k*_c _= 0.634 J s^-1^m^-1^K^-1^, *α *= 2.1 × 10^-4 ^K^-1^. As previously [33], incoming media was assumed to be in equilibrium with atmospheric oxygen and, thus, have an oxygen concentration of *c*_oxy,in _= 0.200 mol m^-3 ^(mM) corresponding to *p*_O2 _≈ 140 mmHg. A number of GSIR perifusion studies including [40] used solutions gassed with enriched oxygen (e.g., 95% O_2 _+ 5% CO_2_; *p*_O2 _≈ 720 mmHg); however, with the islet sizes used here, atmospheric oxygen already provides sufficient oxygenation so that the extra oxygen has no effect on model-calculated insulin secretion (see Results section). Inflow velocity was set to *v*_in _= 10^-4 ^m s^-1 ^(corresponding to a flow rate of 0.1 mL/min in a ~4 mm tube), and along the inlet, a parabolic inflow velocity profile was used: 4*v*_in_*s*(1-*s*), *s *being the boundary segment length.

### Model implementation

The models were implemented in COMSOL Multiphysics 3.5 (formerly FEMLAB; COMSOL Inc., Burlington, MA) and solved as time-dependent (transient) problems allowing intermediate time-steps for the solver. Computations were done with the Pardiso direct solver as linear system solver with an imposed maximum step of 0.5 s, which was needed to not miss changes in the incoming glucose concentrations that could be otherwise overstepped by the solver. With these setting, all computation times were reasonable being about real time; i.e., about 1 h for each perifusion simulations of 1 h interval.

**Figure 3:**
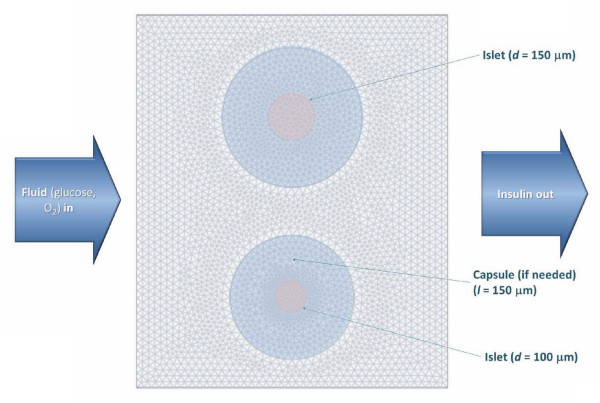
**Geometry and a representative mesh used for the present FEM model**. Two representative spherical islets, which can be either free or encapsulated, are included in a tube with fluid flowing from left to right.

As a representative case, a 2D cross-section of a cylindrical tube with two spherical islets of 100 and 150 μm diameter was used allowing for the possibility of either free or encapsulated islets (capsule thickness *l *= 150 μm; fluid flowing from left to right) (Figure 3). Stepwise increments in the incoming glucose concentration were implemented using again the smoothed Heaviside step function at predefined time points *t_i_*, *c*_gluc _= *c*_low _+ Σ*c*_step,i _flc1hs(*t *- *t_i_*, *τ*). For FEM, COMSOL's predefined 'Extra fine' mesh size was used (5,000-10,000 mesh elements; Figure 3). In the convection and diffusion models, the following boundary conditions were used: insulation/symmetry, **n **(-*D*∇*c+c***u**) = 0, for walls, continuity for islets. For the outflow, convective flux was used for insulin, glucose, and oxygen, **n **(-*D*∇*c*) = 0. For the inflow, inward flux was used for all components with zero for insulin (*N*_0 _= 0), *c*_gluc _*v*_in _for glucose, and *c*_oxy,in _*v*_in _for oxygen. In the incompressible Navier-Stokes model, no slip (**u **= 0) was used along all surfaces corresponding to liquid-solid interfaces. For the outlet, pressure, no viscous stress with *p*_0 _= 0 was imposed.

For visualization of the results, surface plots were used for *c*_ins_, *c*_oxy_, and *R*_ins_. For 3D plots, *c*_ins _was also used as height data. A contour plot (vector with isolevels) was used for *c*_gluc _to highlight the changes in glucose. To characterize fluid flow, arrows and streamlines for the velocity field were also used. Animations were generated with the same settings used for the corresponding graphs. Total insulin secretion as a function of time was visualized using boundary integration for the total flux along the outflow boundary.

## Results and Discussion

### First-and second-phase insulin responses

**Figure 4:**
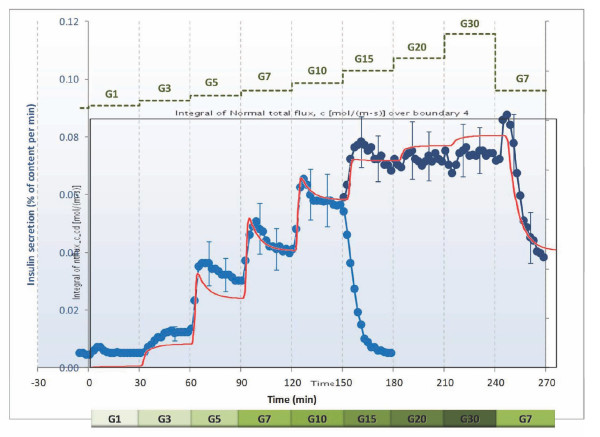
**Glucose-induced insulin secretion in perifused human islets in response to stepwise glucose increments**. Glucose concentration in the perifusing solution increases from 1 mM (G1) to 30 mM (G30) as indicated. Values calculated with the present model (red line, — ) are shown superimposed on the same time-scale over data determined experimentally (blue disks, ●; redrawn from [40]).

**Figure 5:**
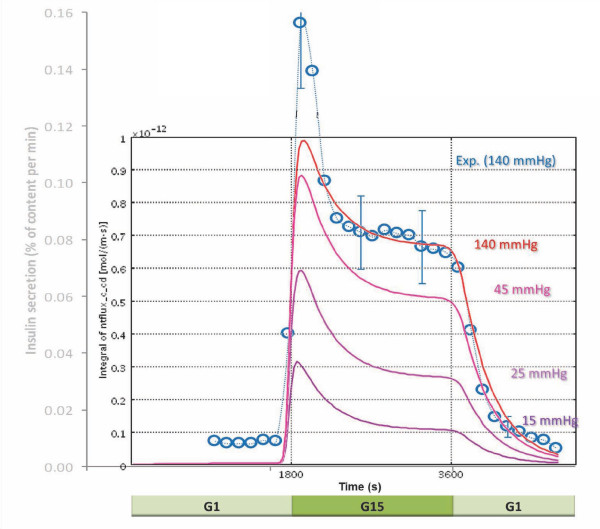
**Oxygen dependence of the calculated GSIR in perifused human islets**. Calculated insulin outflow in response to a stepwise glucose increment from 1 mM (G1) to 15 mM (G15) and back for different incoming oxygen concentrations as indicated by the *p*O_2 _values shown at right. For the normoxic values (red line, —), corresponding values determined experimentally (blue circles, ○; redrawn from [40]) are also shown for comparison.

Following implementation of the model, the values of the adjustable parameters of eqs. 4-9 were selected (Table 1) so as to fit insulin secretion data from islet perifusion experiments with detailed dose responses for glucose-[40,78] and oxygen-dependence [27]. For this purpose, model-predicted insulin responses to stepwise increments in the incoming glucose content were calculated as boundary integrals on the exiting surface of the out-flowing fluid, and these were then fitted to the experimental insulin responses measured as a function of time. First, the parameters of the second-phase response (eq. 6) were fitted to the results of the staircase experiment [40], then those of the first-phase response (eq. 7 and 8). Fine-tuning of the values has been done in a few iterative rounds to also fit the oxygen dependence [27]. As Figure 4 shows, acceptable quantitative agreement can be obtained for both phase 1 and phase 2 responses of the insulin secretion of human islets as measured recently in detailed experiments [40]. The amplitude of the insulin response, which depends on the mass of functional islets present, was adjusted for best fit, but it is within the expected range if calculated for the corresponding number of islet equivalents (IEQ). During the modeling [79], it became apparent that in order to have a correct time-scale and not a very short-term first-phase release, some delay mechanism has to be introduced. After exploring several possibilities, the delay was modeled by incorporation of a 'localized' insulin compartment (e.g., intracellular) from which insulin is then released to the surroundings via first order kinetics (*k*_insL_) (Figure 1). A main reason for this choice was that conceptually, to a good extent, this insulin secreted and stored 'locally' can be considered as the insulin in the readily releasable pool (RRP) of granules in current dynamic cellular models of biphasic insulin secretion [37,65]. It is being filled in response to glucose stimulation (*R*_ins_, eq. 9) and then gradually emptied (*k*_insL_). Our goal at this point, is not to model the detailed cellular and subcellular mechanisms responsible for the biphasic secretion and the staircase response [37,65,66], but to identify functional forms for local responses (*R*_ins_) that when integrated with the descriptions of spatial distribution of the relevant concentrations can give an adequate quantitative description (*c*_gluc_, *c*_oxy _→ *R*_ins _→ *c*_ins_). Most perifusion experiments intended to assess islet quality are performed as single step low-high-low glucose perifusion experiments; a fit for one such data is shown in Figure 5. Again, except for an underestimate of the first-phase peak, acceptable agreement is obtained. First-phase insulin secretion has been shown to be greater following a larger step-up in glucose to the same final value (e.g., both in human [40] and in mouse [80] islets). The present model should account for this as its first-phase insulin secretion rate is determined by the glucose gradient, which increases directly with the size of the step-up; however, some fine-tuning of the parameters is still needed. The predicted first-phase decay (resulting from *k*_insL_) may be a bit too slow (*t*_1/2 _≈ 4 min); however, the prediction of the second-phase decay is more adequate, and to keep the model as simple as possible, we chose to use only one single 'local' insulin 'compartment', hence, a single first-order release rate *k*_insL_.

### Oxygen dependence

**Figure 6:**
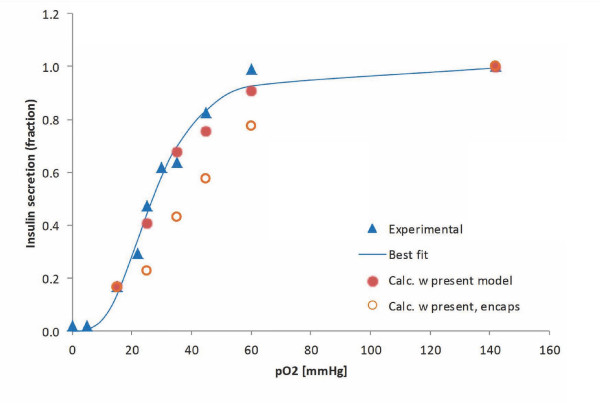
**Influence of oxygen concentration on the insulin secretion rate of perifused islets**. Data represent the fraction of normoxic (second-phase) rate at various oxygen levels in the perifusing media; experimental data (blue triangles) are from [27] and values calculated here are shown as red disks for free islets and orange circles for encapsulated islets. The size-distribution of the islet sample influences these results as larger islets are more severely affected by hypoxia.

**Figure 7:**
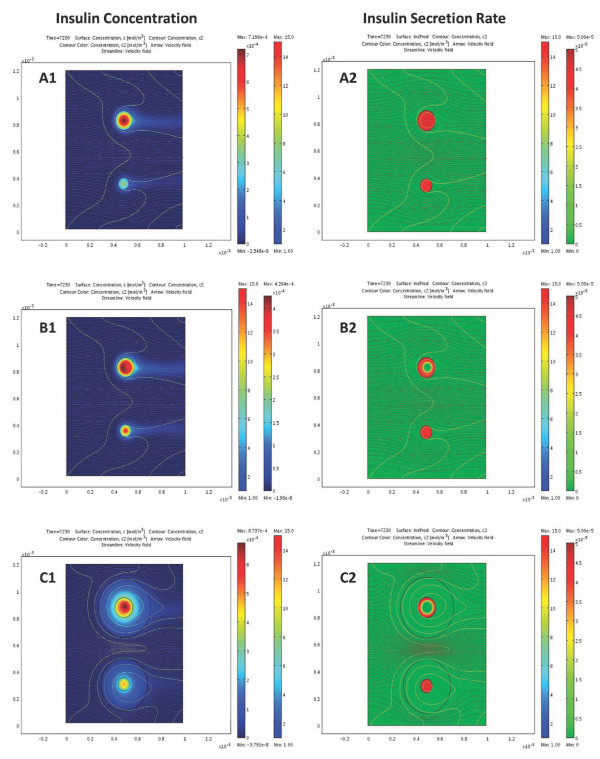
**Comparison of the calculated insulin distribution and secretion rate under various conditions**. Model calculated concentrations during an increase of incoming glucose to 10 mM (corresponding to *t *= 120.5 min in Figure 4) in two perifused islets (*d *= 100 and 150 μm; flow from left to right) under normoxic conditions (**A**), slightly hypoxic conditions (45 mmHg) (**B**), and slightly hypoxic conditions and encapsulation (**C**). Data shown as surface plot are insulin concentration (**1**; shown color-coded from blue for low to red for high-note different scales) and total insulin secretion rate (eq. 9) (**2**; color-coded from green for 0 to red for high and shown on same scale). Gray streamlines and arrows illustrate the velocity field of the flowing perifusion fluid, and colored contour lines show isolevels for the perifusing glucose.

**Figure 8:**
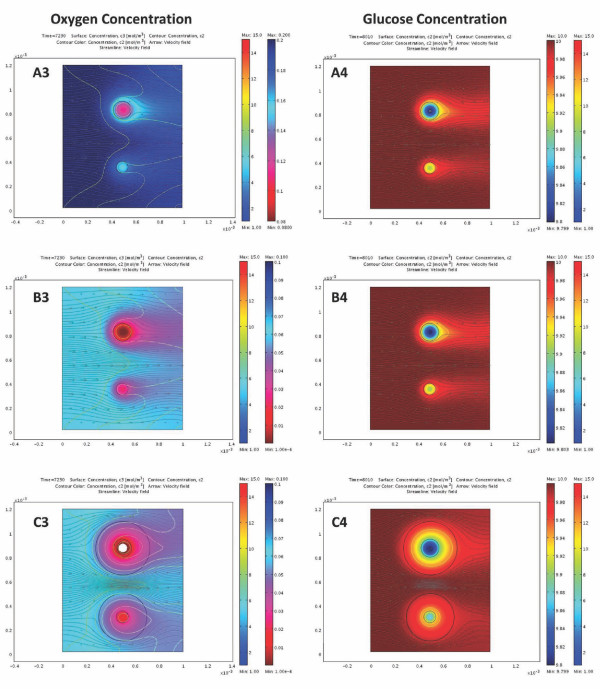
**Comparison of the calculated oxygen and glucose concentrations under various conditions**. Model calculated concentrations in two perifused islets under the same conditions as in Figure 7: normoxic (**A**), slightly hypoxic (45 mmHg) (**B**), and slightly hypoxic and encapsulation (**C**). Data shown as surface plot are oxygen concentration (**3**; shown color-coded from red for low to blue for high with white indicating levels below the critical value *C*_cr,oxy_) and glucose concentration (**4**; shown color-coded from blue for low to red for high). Oxygen concentrations are shown during an increase of incoming glucose to 10 mM (same as in Figure 7); glucose concentrations are shown at a slightly later time point at a constant incoming glucose of 10 mM to avoid the masking effect of the incoming glucose gradient. Note differences in scale between oxygen and glucose, glucose concentrations decreasing by a much smaller percentage than oxygen.

**Figure 9:**
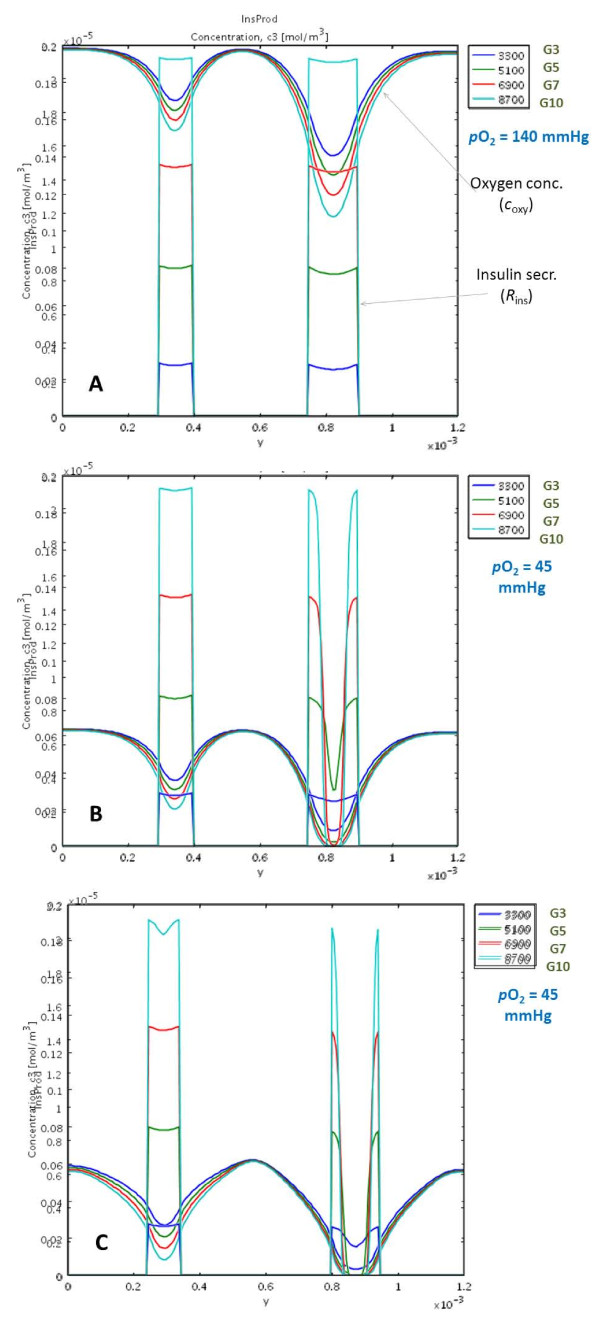
**Comparison of oxygen levels and insulin secretion rates along a vertical cross section**. Calculated values along the vertical mid-section of Figure 3 (or Figure 7) through the two perifused islets under normoxic conditions (atmospheric oxygen, 140 mmHg) (**A**), hypoxic conditions (resembling tissue oxygen concentrations, 45 mmHg) (**B**), and hypoxic conditions and encapsulation (**C**). The two sets of color-coded curves in each figure indicate calculated oxygen concentrations and insulin secretion rates, respectively at various time-points corresponding to glucose concentrations of 3, 5, 7, and 10 mM. At oxygen concentrations that transplanted islets are likely to encounter in their surrounding tissue (~40 mmHg), the insulin secretion of the core region of larger islets is severely compromised.

Because oxygen diffusion is a limiting factor in avascular islets, hypoxia can limit insulin secretion. The oxygen dependence of local insulin release has been parameterized so as to fit the only detailed data available, which, however, are for rat islets [27]. Insulin secretion profiles calculated here for different incoming oxygen concentrations in response to a single glucose step are shown in Figure 5 for a few representative cases. As Figure 6 shows, they give a good quantitative fit of the experimental data of reference [27]. The problem, however, is complicated by the fact that due to the larger diffusion distance, hypoxia is much more severe in the core regions of larger islets [33]; hence, hypoxia will have different effects on the insulin response of differently sized islets. This can be seen in Figure 7 and Figure 8, which show the calculated spatial distributions of insulin, oxygen, and glucose concentrations under normoxic and slightly hypoxic conditions as surface plots. Figure 9 provides further illustration by comparing oxygen concentrations and insulin secretion rates along a vertical cross section of the two islets.

Accordingly, the overall experimental response to hypoxic conditions will depend on the size-distribution of the islet sample. Human islets seem to follow a Weibull distribution with the expected value of islet diameter being around 95 μm and the expected value of islet volume being 1.2 × 10^6 ^μm (corresponding to an islet with *d *= 133 μm) [3]. In other words, most (human) islets are expected to have a diameter around 100 μm, but most of the islet mass (volume) is coming from islets with a diameter around 150 μm, which has been traditionally used as the standard islet (islet equivalent, IEQ) [81,82]. Consequently, we chose two islets with *d *= 100 and 150 μm as representative for our simplified modeling.

It is important to note that even though local insulin release is becoming limited only for oxygen concentrations below 4 mmHg (≈6 μM; eq. 9), the total insulin secretion of the islets starts decreasing rapidly if surrounding oxygen levels drop below ~50 mmHg and is already half-maximal around 25 mmHg (Figure 6). The reason, of course, is that oxygen concentrations in the core of larger islets are considerable less than in the surrounding media due to diffusion limitations (see Figure 8 and 9). It is also worth noting that overall insulin response remains essentially unchanged until oxygen pressures decrease down to ~50 mmHg (Figure 6), values that are present in well vascularized tissues, and then decreases rapidly. This agrees well even with results of *in vivo *experiments in dogs suggesting that moderate hypoxia (*p*_O2 _≈ 40 mmHg) does not affect insulin response, whereas more severe hypoxia (*p*_O2 _≈ 25 mmHg) markedly inhibits it [83]. A number of GSIR perifusion studies including [40] used solutions gassed with enriched oxygen (e.g., 95% O_2 _+ 5% CO_2_; *p*_O2 _≈ 720 mmHg). Compared to atmospheric oxygen (*p*_O2 _≈ 140 mmHg), this does not produces any changes in the insulin profile calculated with the present model (e.g., Figure 4) since with the islet sizes used here atmospheric oxygen already provides sufficient oxygenation so that insulin secretion is not limited (Figure 8A, Figure 9A). On the other hand, transplanted islets are likely to be subject to oxygen levels below 50 mmHg [84] depending on the seeding density and the vascularization of the surrounding tissue, which can further limit their insulin secreting ability. Availability of oxygen is the main limiting factor because, under physiological conditions, oxygen concentrations are considerably lower than glucose concentrations (e.g., around 0.05-0.2 mM vs. 3-15 mM) [62], and this is well illustrated by the present calculation in Figure 8 that compares oxygen and glucose concentrations across the islets. Whereas glucose concentrations in the center of larger islets are only a few percent lower than at the periphery, oxygen concentrations in the center are considerably lower than at the periphery. R. T. Kennedy and co-workers measured somewhat larger glucose concentration decreases in the center of cultured islets (10-20%) [45], but even those are much less severe than the corresponding oxygen decreases.

With the calibrated model, detailed simulations for arbitrary inflow conditions and for arbitrary islet arrangements can be performed, and corresponding detailed graphics and animations can easily be generated. For example, calculated insulin, oxygen, and glucose concentrations along the perifusion chamber with two islets during a glucose gradient are shown in Figure 7 together with the insulin secretion rates. A set of similar results is shown in Figure 9 along a vertical cross-section through the middle of these figures. To illustrate the easy generalizability of the present approach, Additional file 1, Figure S2 shows the results of calculations obtained for a case where a supporting filter was included in the tube. While this perturbs the flow, it has essentially no effect on the overall insulin output justifying the simplifying assumptions made for the present geometry (Figure 3). Increases in the perifusion rate (e.g., up to ten-fold) also have no significant effect on calculated insulin output.

**Figure 10:**
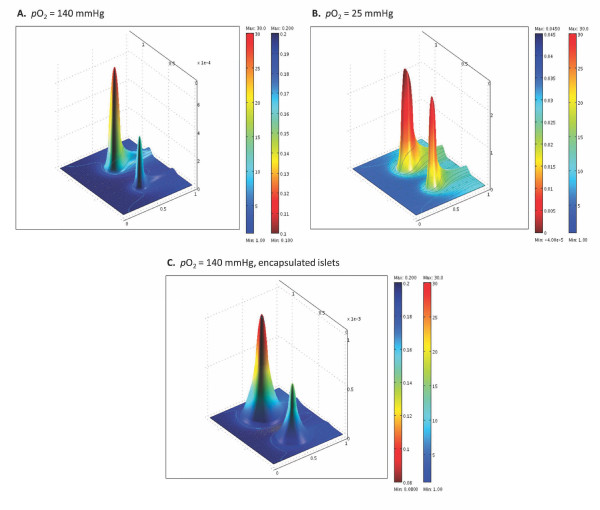
**Calculated insulin concentrations shown as height data**. Surfaces are color-coded for oxygen concentration (blue high, red low) for the same configuration and time-point shown in Figure 7 for free islets in normoxic (*p*_O2 _= 140 mmHg) (**A**) and hypoxic conditions (*p*_O2 _= 25 mmHg) (**B**) as well as for encapsulated islets in normoxic conditions (**C**). The insulin secreting ability of the large islet is more severely affected by hypoxia as clearly indicated by the changes in relative height between **A **and **B **(note different scales).

Figure 10 shows 3D graphs with insulin as height data and color-coded for the oxygen concentrations to further highlight the results of decreasing oxygen concentration in the core region of larger islets. A corresponding set of animations are included as Supporting Information to illustrate the time-course of the first-and second-phase responses following a glucose step (Additional file 2, Video S1; Additional file 3, Video S2). At normoxic conditions (*p*_O2 _= 140 mmHg in the incoming media), the core region of even large islets is still sufficiently oxygenized due to the flowing media; hence, their insulin secretion is not limited. However, this is no longer true for hypoxic conditions as illustrated by Figure 9B or by Figure 10B showing the corresponding results for *p*_O2 _of 45 and 25 mmHg, respectively in the incoming media. Contrary to the previous case, the two islets are predicted to secret about similar insulin amounts despite their different sizes due to the more severe oxygen limitations in the core region of the larger islet, which restricts insulin secretion to an outside shell only in large islets. These results are in good agreement with observations suggesting that smaller islets tend to perform better in islet transplantation (with or without encapsulation) [85-88].

### Encapsulated islets

In patients with type 1 diabetes mellitus, the transplantation of pancreatic islet cells can normalize metabolic control in a way that has been virtually impossible to achieve with exogenous insulin, and is being explored, in a selected cohort of patients with brittle diabetes, as an experimental therapy [89,90]. To avoid the need for life-long immunosuppression, islet encapsulation using semi-permeable membranes and various techniques has long been explored as a possible approach to develop a bioartificial pancreas-an organ capable of releasing insulin in a biomimetic manner in response to plasma glucose changes [11-13]. Many failed attempts [91] made it clear that minimizing the extra volume of encapsulating material (as well as cellular overgrowth) and the corresponding diffusional limitations are crucial for graft success. Hence, there is a considerable interest in modeling the insulin responses of such devices [20-26].

**Figure 11:**
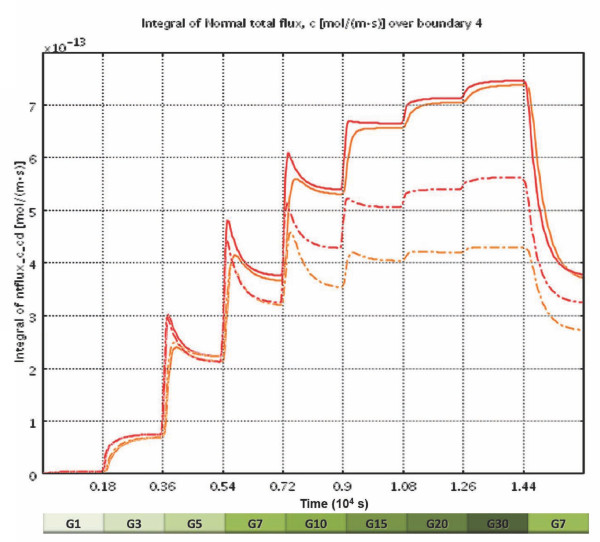
**Calculated GSIR in perifused free vs. encapsulated islets**. Insulin outflow calculated in response to stepwise glucose increments from 1 mM (G1) to 30 mM (G30) under normoxic (continuous line, *p*O_2 _= 140 mmHg) and slightly hypoxic (dashed line; *p*O_2 _= 45 mmHg) conditions for free (red) vs. encapsulated islets (orange). The response of normoxic free islets is the same as given in Figure 4.

Encapsulated islets remain avascular; hence, the present software can be easily extended to model their behavior under perifusion or tissue transplant conditions. For example, as Figure 11 shows, simulations with the present model for microencapsulated islets (assuming hydrogel-like encapsulating material of a relatively modest width, *l *= 150 μm) predicts that perifusion results in somewhat delayed and dampened insulin response, but insulin response kinetics is maintained to a good degree. This is in good agreement with some experimental results obtained, for example, with alginate microencapsulated islets (using oxygen-enriched perifusion media to minimize the effects of oxygen limitation) [92,93]. However, at lower perifusing oxygen concentrations, such as those mimicking tissue oxygen concentrations that transplanted islets are likely to encounter even in well-vascularized tissue (*p*_O2 _= 35-45 mmHg; *c*_oxy _= 0.05-0.065 mM), the loss in insulin secreting ability is much more significant as the encapsulated islets here suffer much more heavily from hypoxia (Figure 7, Figure 9, and Figure 10). Whereas, under these conditions, free islets can still secrete insulin at around 70-75% of their normal rate (and, for transplanted islets, will improve with time as their vasculature is restored), encapsulated islets can only operate at around 50% of the full rate and their response is especially hampered at larger glucose levels (Figure 6, Figure 11). As nicely illustrated by Figure 7 and Figure 9, only a relatively small percentage of the encapsulated islets cells is able to secrete insulin at full capacity; oxygen diffusion limitations severely restrict the hormone secreting ability of the core regions even at this relatively thin microcapsule size-further emphasizing the need for conformal/nano-coating or other alternative approaches [13,94,95]. These results reconfirm the finding of some previous modeling work with cultured or encapsulated islets [75,76], which focused on the limiting effect of hypoxia but without incorporating details of the glucose-insulin response, that also suggested that the use of smaller islets (or islet cell aggregates) could reduce the impact of oxygen diffusion limitation.

### Modeling considerations

Obviously, this is still a much simplified, exploratory model; the actual mechanism of glucose-induced insulin secretion in β-cells is complex and involves various molecular-level events [1,2,36,37,39,70,96,97]. The present model gives an adequate quantitative description of the main distinctive features of insulin release, but, at this stage, does not account for interspecies differences and does not incorporate a number of effects known to affect glucose-induced insulin release including, e.g., amplifiers such as glucagon-like peptide-1 (GLP-1) as well as time-dependent effects (i.e., both time-dependent inhibition and potentiation; e.g., the "glucose priming" effect) [98].

One of the most complex technical control systems that is widely used in industrial control systems and has been suggested as a possibility for the glucose-insulin control system is the proportional-integral-derivative controller (PID controller) [1,8-10]. PID controllers are also particularly promising for closed-loop insulin delivery systems with continuous glucose sensors [8-10,99]. This control loop feedback mechanism uses a combination of proportional, integral, and differential (PID) terms so that the control signal has elements that are functions of the error signal itself (*ε *= *ξ - ξ*_o_, the difference between the existing output *ξ *and its desired value *ξ*_o_), its integral (∫*ε*d*t*), and its differential (d*ε*/d*t*). In other words, the controller output signal *ψ *can be written as a function of time as:(11)

PID control works great in linear systems; however, biological systems are never fully linear: proportional responses are only possible over some limited range as biological responses (e.g., hormone secretion rates, enzymatic degradation rates, nerve firing rates, etc.) are always limited between zero and some maximum value. Biologic systems are always nonlinear, never stationary, but robust, which often comes from coupling of different, overlapping systems [1]. Accordingly, responses in biologic systems are much better described by sigmoid functions; hence, the present suggestion for sigmoid proportional-integral-derivative controllers (SPID controllers) for biologic responses. Use of the Hill function *f*_H _(eq. 2) allows sigmoid responses with limited maximum rates (*R*_max_) and flexible shapes (*n*):(12)

Here, we used the sigmoid direct (proportional) term to model the oxygen and glucose consumptions as well as the second phase insulin release, and the sigmoid differential term to model the first-phase insulin release (with *c*_gluc _itself as the "error" signal *ε*; Figure 1). As always, the role of the differential term is to speed up the system; i.e., to give a large correction signal as soon as possible when the monitored value changes suddenly-exactly the role played by the first-phase insulin secretion. In the present model, we could not yet implement an integral term despite a clear need for such a term over a specified time interval to account, for example, for some inertia and/or delay in insulin secretion (integral control is part of several models, i.e., [8-10,73,99,100]). However, addition of the extra compartment for delayed insulin release actually incorporates some elements usually accounted for by such an integral term.

## Conclusion

In conclusion, a comprehensive insulin secretion model for avascular pancreatic islets has been implemented using Hill-type sigmoid response functions to describe both glucose and oxygen dependence. Detailed spatial distributions of all concentrations of interest are incorporated and coupled via local consumption and release functions. Following parameterization, good fit could be obtained with experimental perifusion data of human islets. Further optimization of the model is required; however, the present approach makes it relatively straightforward to couple arbitrarily complex hormone secretion and nutrient consumption kinetics with diffusive and even convective transport and run simulations with realistic geometries without symmetry or other restrictions-problems that seriously limited previous glucose-insulin modeling attempts. Because of the general framework of the implementation, the model not only helps in the elucidation of the quantitative aspects of the insulin secretion dynamics, but also allows the *in silico *exploration of various conformations involving cultured, perifused, transplanted, or encapsulated islets including the simulation of GSIR perifusion experiments or the study of the performance of bioartificial pancreas type devices.

## Competing interests

The author declares that he has no competing interests.

## Authors' contributions

PB is the only author.

## Supplementary Material

Additional file 1 **Supporting Information, Figures S1 and S2**. Two supporting figures with Figure S1 showing the local oxygen-dependent modulating function and Figure S2 showing model calculations with a supporting filter included in the perifusion tube. Click here for file

Additional file 2 **Supporting Information, Video S1**. Movie file showing the time-course of the insulin response of two islets to a glucose step (3 mM → 11 mM → 3 mM) under normoxic conditions (*p*O_2 _140 mmHg) in a 3D representation with insulin concentration as height data and a surface color-coded for oxygen concentration (similar to Figure 10). Click here for file

Additional file 3 **Supporting Information, Video S2**. Movie file showing the time-course of the insulin response of two islets to a glucose step (3 mM → 11 mM → 3 mM) under hypoxic conditions (*p*O_2 _25 mmHg) in a 3D representation with insulin concentration as height data and a surface color-coded for oxygen concentration (similar to Figure 10). Click here for file
