# Supplementary material for: A local glucose-and oxygen concentration-based insulin secretion model for pancreatic islets
Source: Theor Biol Med Model. 2011 Jun 21;8:20. doi: 10.1186/1742-4682-8-20 (PMC3138450; doi:10.1186/1742-4682-8-20)
Supplement: Additional file 1 — Supporting Information, Figures S1 and S2. Two supporting figures with Figure S1 showing the local oxygen-dependent modulating function and Figure S2 showing model calculations with a supporting filter included in the perifusion tube. [file 1742-4682-8-20-S1.PDF]

Supporting Information

Video S1: GlucInsDyn_wDel_Model05_G3G11.wmv

Movie file showing the time-course of the insulin response of two islets to a glucose step (3 mM $\rightarrow$ 11 mM $\rightarrow$ 3 mM) under normoxic conditions (pO_2 140 mmHg) in a 3D representation with insulin concentration as height data and a surface color-coded for oxygen concentration (similar to Figure 10).

Video S2: GlucInsDyn_wDel_Model05_G3G11_25mmHg.wmv

Movie file showing the time-course of the insulin response of two islets to a glucose step (3 mM $\rightarrow$ 11 mM $\rightarrow$ 3 mM) under hypoxic conditions (pO_2 25 mmHg) in a 3D representation with insulin concentration as height data and a surface color-coded for oxygen concentration (similar to Figure 10).

Supporting Information – Figures

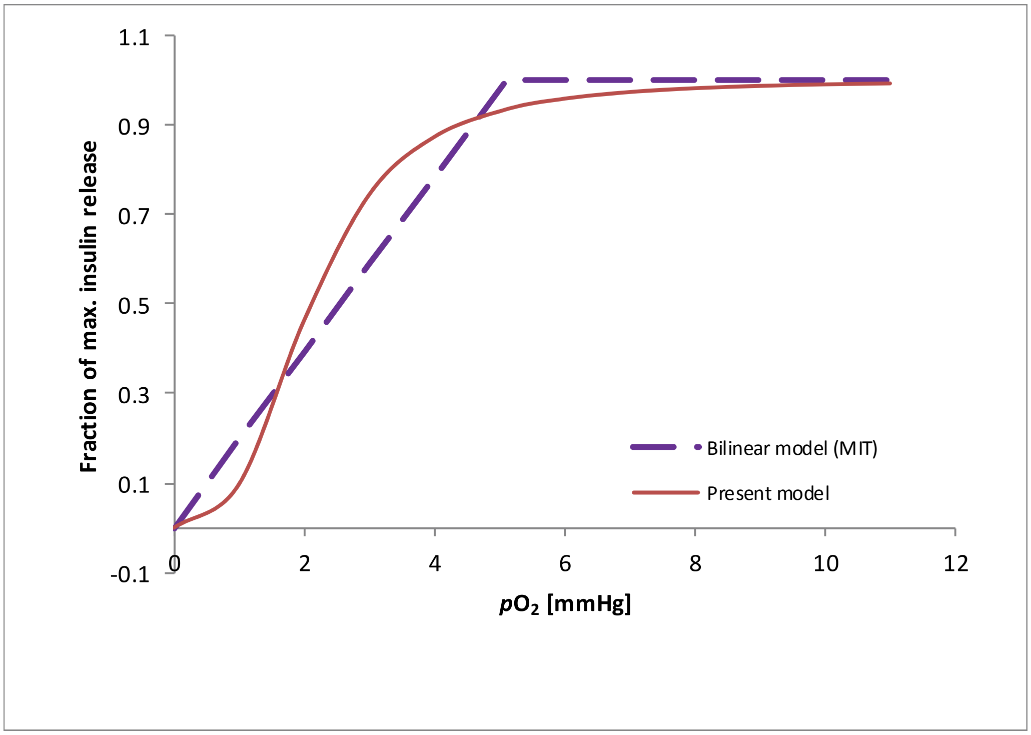

Figure S1. **The local oxygen-dependent modulating function.** The modulating function, $\varphi_{i,o}(c_{oxy})$, used in the present model (eq. 9; $n_{ins,oxy}$ = 3, $C_{Hf,ins,oxy}$ = 3 μM → $p_{Hf,ins,oxy}$ = 2 mmHg) to limit insulin secretion at low oxygen concentrations (dark red line, —) and the bilinear function used by Colton and co-workers [76, 77] for similar purposes (dashed purple line, – –).

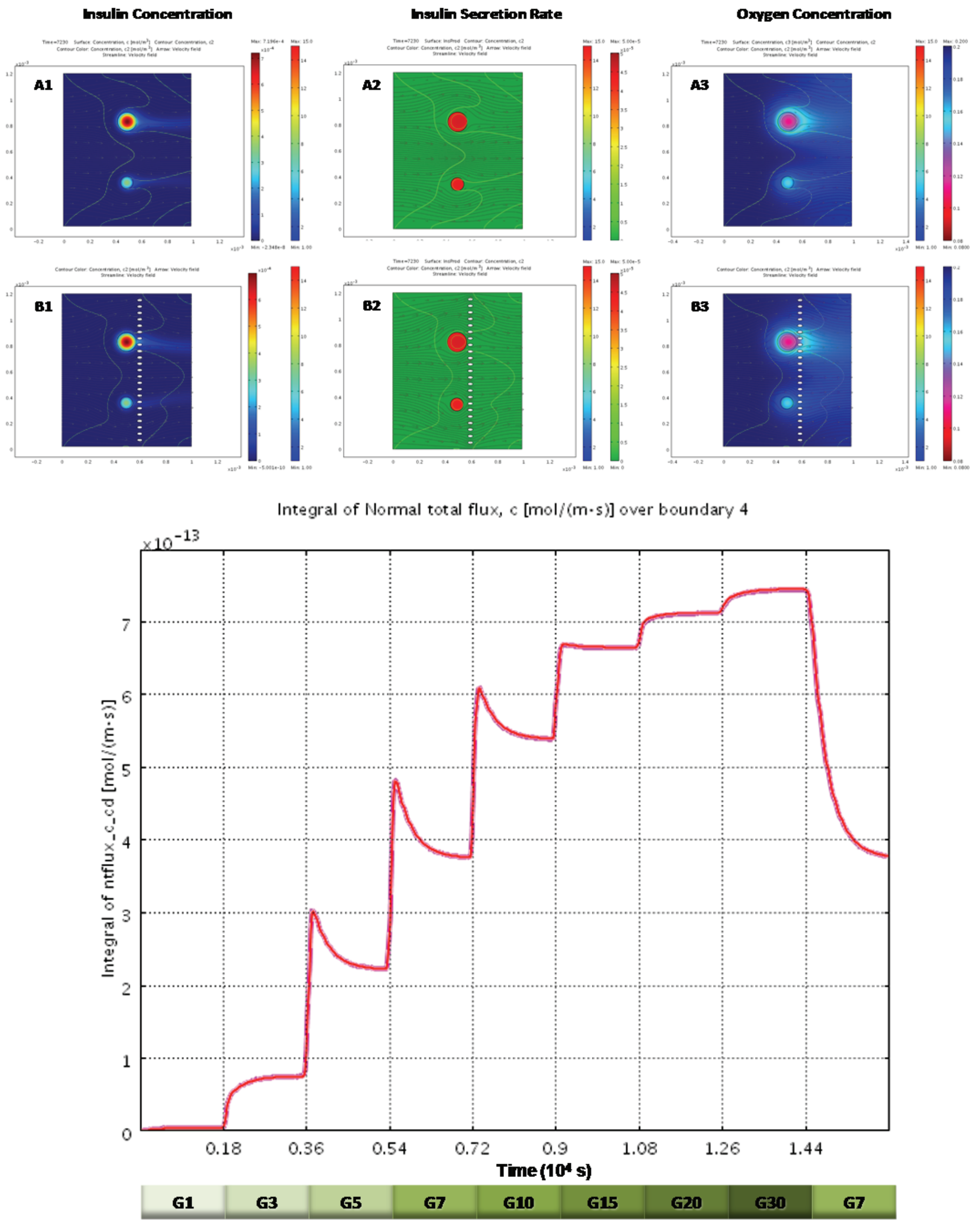

Figure S2. **Model calculations with a supporting filter included in the perifusion tube**. Inclusion of a filter perturbs the flow (top figures, **A** vs. **B** with the row **A** being the same as in Figure 7), but has no effect on the overall insulin output (bottom figure showing overlapping graphs calculated for the same condition as Figure 4 that are essentially indistinguishable; red line, —: configuration **A** without filter, thicker magenta line, —: configuration **B** with filter).
